# *GSTM1* and Liver Iron Content in Children with Sickle Cell Anemia and Iron Overload

**DOI:** 10.3390/jcm8111878

**Published:** 2019-11-05

**Authors:** Latika Puri, Jonathan M. Flanagan, Guolian Kang, Juan Ding, Wenjian Bi, Beth M. McCarville, Ralf B. Loeffler, Aaryani Tipirneni-Sajja, Martha Villavicencio, Kristine R. Crews, Claudia M. Hillenbrand, Jane S. Hankins

**Affiliations:** 1 Department of Hematology, St Jude Children’s Research Hospital, Memphis, TN 38105, USA; martha.villavicencio@stjude.org (M.V.); jane.hankins@stjude.org (J.S.H.); 2 Division of Hematology, Department of Pediatrics, Baylor College of Medicine, Houston, TX 77030, USA; jmflanag@txch.org; 3 Department of Biostatistics, St Jude Children’s Research Hospital, Memphis, TN 38105, USA; guolian.kang@stjude.org (G.K.); juan.ding@stjude.org (J.D.); 4 Department of Biostatistics and Center for Statistical Genetics, University of Michigan, Ann Arbor, MI 48109, USA; 5 Department of Diagnostic Imaging, St Jude Children’s Research Hospital, Memphis, TN 38105, USA; beth.mccarville@stjude.org; 6 Department of Research Imaging, University of New South Wales, Randwick, NSW 2052, Australia; ralf.loeffler@unsw.edu.au (R.B.L.); claudia.hillenbrand@unsw.edu.au (C.M.H.); 7 Department of Biomedical Engineering, University of Memphis, Memphis, TN 38105, USA; aaryani.sajja@memphis.edu; 8 Department of Pharmaceutical Sciences, St Jude Children’s Research Hospital, Memphis, TN 38105, USA; kristine.crews@stjude.org

**Keywords:** sickle cell anemia, iron overload, glutathione S-transferase M1 (GSTM1), chelation therapy

## Abstract

Chronic blood transfusions in patients with sickle cell anemia (SCA) cause iron overload, which occurs with a degree of interpatient variability in serum ferritin and liver iron content (LIC). Reasons for this variability are unclear and may be influenced by genes that regulate iron metabolism. We evaluated the association of the copy number of the glutathione S-transferase M1 (*GSTM1*) gene and degree of iron overload among patients with SCA. We compared LIC in 38 children with SCA and ≥12 lifetime erythrocyte transfusions stratified by *GSTM1* genotype. Baseline LIC was measured using magnetic resonance imaging (MRI), R2*MRI within 3 months prior to, and again after, starting iron unloading therapy. After controlling for weight-corrected transfusion burden (mL/kg) and splenectomy, mean pre-chelation LIC (mg/g dry liver dry weight) was similar in all groups: *GSTM1* wild-type (WT) (11.45, SD±6.8), heterozygous (8.2, SD±4.52), and homozygous *GSTM1* deletion (*GSTM1*-null; 7.8, SD±6.9, *p* = 0.09). However, after >12 months of chelation, *GSTM1*-null genotype subjects had the least decrease in LIC compared to non-null genotype subjects (mean LIC change for *GSTM1*-null = 0.1 (SD±3.3); versus −0.3 (SD±3.0) and −1.9 (SD±4.9) mg/g liver dry weight for heterozygous and WT, respectively, *p* = 0.047). *GSTM1* homozygous deletion may prevent effective chelation in children with SCA and iron overload.

## 1. Background

Chronic blood transfusion therapy is frequently used for both primary and secondary stroke prevention in children with sickle cell anemia (SCA) [1,2]. Iron overload is an inevitable complication of chronic erythrocyte transfusions because there is no physiological mechanism to eliminate the exogenous iron introduced by transfused erythrocytes. Iron deposits in target organs, including the liver and heart, cause organ dysfunction and, if untreated, may lead to organ failure [3,4]. Iron overload can be a significant problem in SCA [5]. Complications from iron damage are responsible for up to 11% deaths in patients with SCA [6] and therefore must be carefully monitored and treated [3]. 

In SCA, iron overload occurs with a degree of interpatient variability in both the serum ferritin [7] and liver iron content (LIC) [8,9,10]. The reasons for this interpatient variability are not completely understood, but genetic modifiers could represent an important source of such variability in iron accumulation. In our prior work, several candidate genes were found to be significantly differentially expressed in a group of SCA patients with iron overload [11]. Among these genes found in an extreme phenotype analysis, average expression of glutathione S-transferase M1 (*GSTM1*) in the liver was found to be almost completely absent in patients with high LIC (>18.5 mg Fe/g dry weight liver) compared to patients with lower LIC (<10.5 mg Fe/g dry weight liver) [11]. We discovered that the reason for this significant reduction in *GSTM1* expression (*p* < 10^−60^) was likely due to a polymorphic whole gene deletion of the *GSTM1* gene. Subjects with a homozygous deletion of the *GSTM1* locus (*GSTM1*-null; GSTM1*0) have no functional enzymatic activity. In our sample, the *GSTM1*-null genotype was more prevalent in SCA patients in the high LIC group compared to the low LIC group (5 out of 7 patients vs. 5 out of 18 patients, *p* = 0.017) [11]. This suggested that decreased *GSTM1* expression was either associated with increased iron loading in the liver or resistance to iron unloading in response to chelation efforts in SCA patients with similar transfusion burden [11]. 

*GSTM1* plays an important role in detoxification of carcinogens, therapeutic drugs, and products of oxidative stress, by conjugation with glutathione. Importantly, *GSTM1* deletion has been associated with increased cardiac iron in thalassemia major [12] and increased disease severity in patients with SCA [13]. However, prior studies of the *GSTM1* genotype in SCA have not prospectively evaluated its association with iron loading and unloading during chelation therapy, nor did they control for iron chelation exposure during iron burden assessment. Understanding of modifiers of iron chelation therapy may allow tailoring of management to specific genetic profiles. Based on our previous findings, we aimed at validating the relationship of *GSTM1* deletion with liver iron burden in an independent prospectively collected sample. We tested the hypothesis that *GSTM1* homozygous gene deletion was associated with higher pre-treatment LIC, as measured by hepatic magnetic resonance imaging (MRI), R2*MRI, in a longitudinal cohort of children with SCA who initiated transfusions for primary or secondary stroke prevention. As secondary objectives, we explored the association of *GSTM1* genotypes with LIC R2*MRI during chelation, serum ferritin, and heart T2*MRI values. 

## 2. Methods 

### 2.1. Patient Selection

Participants were included in this single-site prospective longitudinal observational cohort study (Genes Influencing Iron Overload State, GENIOS, NCT01158794) if they had SCA (HbSS or HbSβ^0^-thalassemia) and had received ≥12 lifetime erythrocyte transfusions (simple red cell transfusions or erythrocytapheresis). Additionally, patients were eligible only if they had a baseline LIC assessment, which was defined as an LIC obtained by R2*MRI within 3 months prior to initiation of iron chelation therapy. Year 1 LIC was defined as an LIC obtained at least 12 months after initiation of iron unloading therapy (iron chelation or therapeutic phlebotomy). The enrollment period lasted for 3 years. Our cohort design ensured the uniform assessment of tissue iron loading and unloading prior to and during iron chelation, respectively. All participants received erythrocyte transfusions for primary or secondary stroke prevention. Subjects were recruited during routine clinic or transfusion visits. The study was approved by the St. Jude Children’s Research Hospital IRB, and informed consent was obtained from all participants or their legal guardian (if participants were minors). 

### 2.2. Data Collection

The complete transfusion and chelation history of all participants was retrospectively abstracted and then prospectively collected after enrollment, including volume of blood transfused, pre-transfusion weight, chelator agent(s), dose(s), dose adjustments, reasons for dose changes, and chelation adverse effects. Adherence data for iron chelation therapy was estimated by calculation of medication possession ratio (MPR) as follows: amount of drug dispensed (days)/interval between dispensations (days). The MPR was considered adequate at ≥80%, as previously reported [14,15,16,17]. History of splenectomy was also collected. 

### 2.3. MRI Studies

Liver R2*MRI and heart T2*MRI were obtained after at least 12 erythrocyte transfusions and within three months prior to iron unloading therapy initiation (baseline assessment). They were then repeated at least 12 months after the start of iron unloading therapy (Year 1 assessment). The R2*MRI scan was obtained on a 1.5T MRI scanner (Siemens Symphony, Siemens; Malvern, PA) using a multi-echo gradient echo sequence acquiring 20 images with increasing echo times (range, 1.1–17.3 ms). Liver images were obtained in transversal slice orientation through the center at the main portal vein origin with a slice thickness of 10 mm and in-plane resolution of 3.125 mm. Quantitative T2* maps were calculated offline using custom-written MATLAB software (MathWorks, Natick, MA), and signal intensity drop was fitted on a pixel-by-pixel basis to a mono-exponential decay using a least-squares fit method. The used fitting method treats noise in the model by measuring and accounting for image noise [18]. Regions of interest (ROIs) were drawn either on source images or T2* maps and included the entire axial cross-section of the liver. Vessels and other structures that would affect the mean liver R2* value were removed via a T2* histogram analysis as previously described [19,20]. T2* was converted to its reciprocal R2* using the formula: R2*(Hz) = 1000/T2*(ms). LIC was estimated in mg/g of liver dry weight using a calibration curve as previously described. 

For assessment of myocardial iron content, an adequate ROI was identified on the calculated T2* map in the left ventricular septum, distant from the lungs and cardiac veins, which are known to cause susceptibility artifacts. This ROI encompassed both endocardial and epicardial regions, where most of the myocardial iron is accumulated [21,22]. The area size of the ROI was kept as uniform as possible from patient to patient. The mean T2* value obtained from cardiac ROIs was calculated for each participant. T2* values below 20 ms were considered as elevated due to their relationship with cardiac complications.

### 2.4. GSTM1 Assay

Genomic DNA for *GSTM1* genotyping was isolated from peripheral blood from all patients. A copy number assay for *GSTM1* (NM_000561) was performed using a genomic DNA real-time PCR procedure. The quantitative real-time PCR was performed on an Applied Biosystems StepONE instrument (AB, Foster City, CA, USA). The *GSTM1* locus was amplified using a commercially available assay (USA; Hs00273142_cn) in a multiplexed PCR containing an internal standard copy number PCR. The *GSTM1* copy number of all samples was then quantified relative to the internal standard two-copy gene control RNase P. 

### 2.5. Statistical Analysis

The primary objective of the study was to investigate the association of *GSTM1* gene deletion and the LIC pre-iron unloading therapy (chelation and therapeutic phlebotomy) among patients with SCA and transfusional iron overload. Our preliminary data suggested that there was no interaction between *GSTM1* genotypes and the transfusion duration nor a significant difference between *GSTM1* genotypes with one and two copies of the gene (i.e., *GSTM1* heterozygous deletion and wild-type genotypes) [11]. Therefore, patients were considered at risk if they had a *GSTM1*-null genotype (homozygous deletion). The powers were calculated using PROC GLMPOWER from SAS (version 9.2) with one-way analysis of variance (ANOVA) adjusted by covariates. With a sample size of 38 the power to detect a significant association between LIC and *GSTM1* genotypes was 78%, assuming two means of 11.432 mg/g liver dry weight and 5.707 mg/g dry liver dry weight, a common standard deviation of 6 mg/g liver dry weight at a significance level of 0.05 with a two-group sample size ratio of 2, and a correlation between response and covariate of 0.5. The primary outcome of LIC was controlled by the lifetime cumulative transfusion burden corrected by weight (in mL/kg). 

Summary statistics were reported as median, standard deviation and range and the number and frequency for continuous and categorical variables and were compared using Kruskal–Wallis or ANOVA test and Fisher’s exact test, respectively. Shapiro-Wilk normality test was used to test the normality of data. We first checked the recessive model per study design and interaction between genotype and chelation duration. We then considered three other genetic models, the additive model, dominant model, and co-dominant model, to test if there were significant associations under different genetic models. Box-cox transformation was used to transform data if data did not follow normal distribution. Univariate and multivariate linear regression analyses were used to assess the associations between outcomes and covariates. Similar analysis was performed for serum ferritin and heart T2*MRI. MPR was calculated monthly and averaged for the interval between baseline and Year 1. All *p*-values were two-sided and considered statistically significant if <0.05. All analyses were done using R-3.6.1.

## 3. Results

### 3.1. Patient Characteristics

Thirty-eight children with SCA with a median age of 7.4 years (range 3.5–18 years) were enrolled and prospectively followed. Characteristics of the patient population are described in Table 1. The distribution of *GSTM1* genotype group was: homozygous deletion (*n* = 7), heterozygous (*n* = 19), and wild type (WT) (*n* = 12). There were no significant differences in patient demographics, transfusion volume, type of chelation therapy, and splenectomy among the three *GSTM1* genotype groups (Table 1). All 38 patients received simple transfusions as the primary method of transfusion therapy, however, 15 of them received erythrocytapheresis for a brief period of time during the observation period. The mode of transfusion was similar in the three groups (Table 1). The mean transfusion burden up to baseline and upon iron chelation initiation was not different among the three groups (Table 1).

Thirty-five (92%) subjects initiated iron chelation at a mean duration of 1.5 months from the baseline liver R2*MRI; most patients (*n* = 32) received deferasirox for iron chelation, and the remaining received deferoxamine (*n* = 1), a combination of deferasirox and deferoxamine (*n* = 1), or therapeutic phlebotomy (*n* = 1). Baseline cardiac T2* was available for 23 patients (*GSTM1* homozygous deletion, *n* = 5; *GSTM1* heterozygous deletion, *n* = 11; and *GSTM1* WT, *n* = 7). MPR data were available for 28 patients. 

### 3.2. Liver Iron Content (LIC) and GSTM1 Genotype

**Figure 1:**
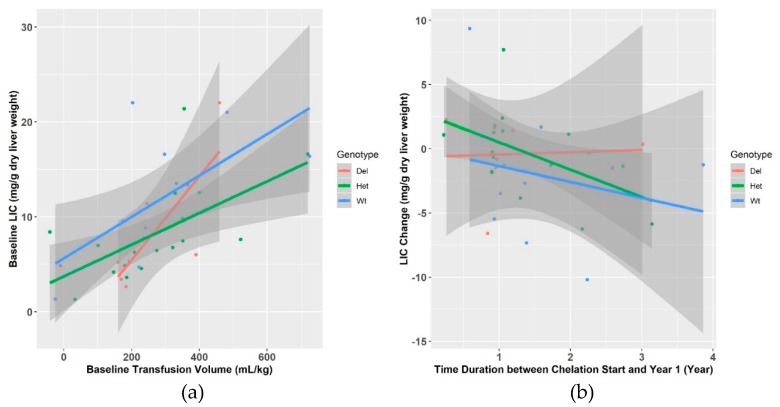
Liver iron content (LIC) R2*MRI differences according to glutathione S-transferase M1 *(GSTM1*) genotype groups. (**a**) LIC increases with transfusion burden. Baseline LIC is variable for similar transfusion burden and not significantly different by *GSTM1* genotype (*p* = 0.09). (**b**) During iron unloading therapy (chelation and therapeutic phlebotomy), patients with homozygous *GSTM-1* deletion had higher LIC than those with heterozygous and wild type genotypes (*p* = 0.047).

We first measured LIC at baseline, prior to the initiation of iron unloading therapy. The overall mean LIC for the entire cohort was 9.1 (SD:5.9) mg/g of liver dry weight. The mean LIC was not statistically significantly higher in the WT group (11.45 mg/g of liver dry weight, SD±6.8) as compared to the heterozygous (8.2 mg/g of liver dry weight, SD±4.5) and homozygous deletion (7.8 mg/g of liver dry weight, SD±6.9) groups (*p* = 0.22). This difference was marginally significant in multivariate analysis that controlled for transfusion burden and splenectomy under a dominant model (*p =* 0.09) (Table 2, Figure 1a) but not under the other genetic models. 

The mean duration of treatment for the 35 children who received iron unloading therapy was 17.8 months and it was not different among the three *GSTM1* genotype groups (Table 2). The mean MPR for the entire group was 81% (SD±16) and was similar among the three genotype groups (homozygous deletion: 83%, SD±19%; heterozygous deletion: 82%, SD±15%; WT genotype: 78%, SD±17%, *p =* 0.845). 

The mean change in LIC at Year 1 was the smallest for the *GSTM1*-null genotype as compared to the non-null genotype groups (mean change for *GSTM1*-null genotype = 0.1 (SD±3.3); *GSTM1* heterozygous deletion = −0.3 (SD±3.2); and WT: −1.9 (SD±4.9) mg/g liver dry weight, Table 2). In a multivariate analysis, controlling for transfusion burden, duration of chelation, interaction of chelation and genotype, and presence of splenectomy, the degree of change in LIC was lowest in subjects with *GSTM1*-null genotype as compared to those with non-null genotypes (*p =* 0.047) (Figure 1b). 

### 3.3. Serum Ferritin and GSTM1 Genotype

Mean serum ferritin levels were also evaluated at baseline and Year 1 in a similar fashion to LIC. Serum ferritin at baseline for the entire cohort was 2879.7 ng/dL (SD±1528). Like LIC, serum ferritin was the highest in the *GSTM1* WT genotype (3335.3 ng/dL, SD±2016 ng/dL) followed by the heterozygous genotype (2763.6 ng/dL, SD±1284.5 ng/dL) and the *GSTM-*null genotype (2413.6 ng/dL, SD±1133.7), but was not significantly different (Table 2, Supplemental Figure S1a). In multivariate analysis controlling for transfusion burden and splenectomy, this difference was not significant for all genetic models considered (*p* = 0.29). At Year 1 there was an overall increase in ferritin in all three groups. The mean increase in serum ferritin for the entire cohort was 250.6 ng/dL (SD±1130 ng/dL). The mean increase in serum ferritin was the lowest in the WT group (31.92 ng/dL, SD±1204.5 ng/dL) followed by the *GSTM1* homozygous deletion group (162.6 ng/dL, SD±845.4 ng/dL) and was the greatest for the *GSTM1* heterozygous group (421.1 ng/dL, SD±845.4 ng/dL), but was not significantly different (Table 2, Supplemental Figure S1b). In multivariate linear regression analysis controlling for transfusion burden, splenectomy, chelation duration and MPR, this change in ferritin was not statistically different among the three groups (*p* = 0.11).

### 3.4. Cardiac Tissue Iron and GSTM1 Genotype

Cardiac MRI T2* was measured at baseline in 23 out of the 38 participants. Of these 23 participants, elevated cardiac iron was noted in only one participant, a child with the heterozygous *GSTM1* deletion. This patient had a baseline heart T2* of 19.6 ms that improved to 41.1 ms at Year 1. Pre-chelation baseline mean T2* value for the entire cohort was 37.5 ms (SD±6.7 ms) and it was similar across the three *GSTM1* genotype groups (Table 2; Supplemental Figure S2a). This difference remained non-significant for four genetic models in multivariate analysis controlling for transfusion burden and splenectomy (*p* = 0.86). After iron unloading therapy was initiated, cardiac T2* increased but the change was not statistically different among the three *GSTM1* genotype groups in multivariate linear regression analysis, controlling for transfusion burden, splenectomy and chelation duration (change in in T2* for the *GSTM1* homozygous deletion group: 3.9 ms (SD±2.58 ms); *GSTM1* heterozygous deletion group: 0.95 ms (SD±7.9.17 ms); WT group: −1.25 ms (SD±3.18 ms); *p* = 0.2) (Table 2, Supplemental Figure S2b). 

## 4. Discussion

Chronic blood transfusion plays a vital role in the primary and secondary prevention of stroke in patients with SCA but leads to significant iron overload. Chronically transfused patients accumulate iron at a rate of 0.3 to 0.4 mg/kg/day, but with a large degree of inter-individual variability. In our cohort of children with SCA who received monthly erythrocyte transfusions and were prospectively followed during the iron loading and unloading phases of treatment, we found that baseline LIC was not significantly different in the three genotype groups. However, patients with the *GSTM1*-null genotype seemed to have unloaded iron slowly as compared to the nonnull genotypes.

During chronic transfusion therapy, there is inter-patient variability in both the serum ferritin [7] and the LIC [8,9,10]. This variability was first noted in a prospective study that evaluated longitudinal changes in the serum ferritin of 50 children with SCA treated with chronic transfusion [7]. This study noted a remarkable interpatient variability in serum ferritin. About 10% of the patients were labelled as rapid risers due to a steep increase in serum ferritin levels after relatively small transfused blood volumes. The same degree of variability has also been noted in LIC when similar durations of blood transfusion were considered [8,9,10].

The reasons for such variability of iron overload in response to chronic blood transfusions are not completely understood. The presence of the spleen seems to provide a “buffering effect” by avoiding iron accumulation in preferential organs, such as the heart and liver, in exchange for a greater splenic iron deposition. This could explain why the LIC values vary among patients with and without their spleen. However, it was demonstrated that the presence of the spleen was not a likely explanation for five of fifty patients with a rapid rise in serum ferritin in a longitudinal study of patients with SCD who received transfusions for a mean of 39 months [7]. This subgroup of five patients did not differ in terms of volume of blood transfused or type of transfusion received from patients who had a slow ferritin rise and were not splenectomized, suggesting that there are other reasons for variability in iron overload. Our findings are in agreement with the literature and we did not see a modifying effect of splenectomy in the relationship between *GSTM-1* and LIC.

Our study is one of the few to prospectively follow a cohort of patient with iron-overload and determine the relationship between iron accumulation and removal with genetic polymorphisms in *GSTM1*. In our study, we noted that after controlling for transfusion burden and splenectomy, iron loading (baseline LIC) varied greatly in our patients (Figure 1a). In contrast with our previous findings, we found that patients homozygous *GSTM1* deletion had lower baseline LIC as compared to the wild type genotypes. After chelation was initiated, patients with the *GSTM1*-null genotype unloaded iron more slowly as compared to *GSTM1* nonnull genotypes. This indicates that homozygous deletion of *GSTM1* may negatively impact iron unloading, suggesting that the association of *GSTM1* with higher LIC may reflect the impact of *GSTM1* expression on the efficacy of chelation therapy. In our previous cross sectional study, patients with the homozygous *GSTM1* deletion had higher LIC as compared to heterozygote or wild type patients, but all of these patients were already on chelation [11]. Thus, the initial association of the *GSTM1* deletion with high LIC could reflect the resistance to iron chelation in patients with the *GSTM1* homozygous deletion, as most SCA patients in our previous study had at least some history of iron chelation treatment. 

In the present study, we have comprehensively measured the degree of iron loading and controlled for individual transfusion burden, presence or absence of splenectomy, duration of chelation, patient’s adherence to chelation treatment, and assessed response to chelation by measuring changes in LIC over time. We have found that *GSTM1* gene deletion was associated with decreased iron unloading, where patients with the *GSTM1-*null genotype had the least change in LIC, heterozygotes had an intermediate rate of iron unloading, and wild type patients had the biggest reductions in LIC (Figure 1b). Similar trends were observed in serum ferritin, such that individuals with *GSTM1* homozygous deletion had least increase in serum ferritin values after iron unloading treatment compared to the other two genotypes. However, serum ferritin has limitations in SCD as an accurate marker of iron overload due to ongoing chronic inflammation [23]. Taken together, our prior and current work suggest that the polymorphic *GSTM1* gene deletion results in reduced *GSTM1* gene expression and this may be associated with delays in iron unload during chelation therapy.

The *GSTM1* enzyme functions to protect cells and tissue from oxidative stress, as well as carcinogens, medications and environmental toxins, by conjugation with glutathione [13]. The *GSTM1* gene is highly polymorphic, with the inherited null genotype increasing an individual’s susceptibility to carcinogens and toxins as well as affecting the toxicity and efficacy of certain drugs. Excessive iron overload can also result in oxidative stress. Thus, patients with the *GSTM1* deletion, who may not unload iron effectively, could be at increased susceptibility to oxidative stress which could lead to more organ damage. 

Various genes play a role in regulation of iron and can be explored in a similar way to better understand the genetic modifiers that could impact iron overload in patients who receive chronic transfusion. Future work investigating different genes regulating iron metabolism will help guide choice and dose of chelation therapy for these patients.

Glutathione-S transferase gene deletions (*GSTM1* and *GSTT 1*) have also been associated with increased cardiac iron deposition in patents with thalassemia who are receiving chronic transfusion therapy [12,24,25,26]. In our study population we did not observe severe cardiac iron overload as measured by cardiac T2* in the majority of participants and their T2* values were similar among all three groups. This is consistent with previous studies where severe cardiac iron overload has not been noted despite high liver iron burden in patients with SCA [24,27].

Some limitations of our study warrant discussion. The study population is small and iron chelation adherence data were not available for all participants, so our findings need to be validated in a larger population. Our small sample might have precluded us from seeing significant differences in both heart T2* and serum ferritin among the three groups. Additionally, our follow up period is only 21 months after iron unloading therapy initiation, and therefore long-term follow up is necessary to evaluate if LIC changes will remain different when stratified by *GSTM1* genotypes. If our results are replicated in a larger sample, treatment for iron overload with chelators in patients with sickle cell disease should be individualized for the GSTM-1 status, such that patients with homozygous deletion for GSTM-1 should receive a more aggressive iron unloading treatment (e.g., dual therapy and maximized dosing). 

## 5. Conclusions

There is interpatient variability in development of iron overload in patients with SCA who receive chronic transfusion therapy. Their response to chelation therapy is also variable. Homozygous deletion of *GSTM1* may interfere with iron chelation therapy and lead to slow unloading of liver iron. These findings need to be confirmed in a larger cohort to help inform chelation therapy for patients with SCA. 

## Figures and Tables

**Table 1:** Characteristics of study participants.

Demographic Variables	Total (*n* = 38)	*GSTM1* Homozygotes (*n* = 7)	*GSTM1* Heterozygotes (*n* = 19)	*GSTM1* Wild Type (*n* = 12)	*p*-Value
**Demographics**					
**Median age at baseline (Range) (Years)**	7.5 (3.5–18.1)	5.84 (4.7–18.1)	7.6 (3.47–15.8)	8.5 (4.0–17.3)	0.6
**Sex (Male/Female)**	15/23	1/6	7/12	7/5	0.2
**Sickle genotype (HbSS/HbSβ^0^-thalassemia)**	37/1	7/0	18/1	12/0	1
**Transfusion Regimen**					
**Simple transfusions (%)**	23 (60.5)	4 (57.1)	14 (73.7)	5 (41.7)	0.2
**Simple and Exchange transfusions (%)**	15 (39.5)	3 (42.9)	5 (26.3)	7 (58.3)	0.2
**Mean Cumulative Blood Transfusion Volume**					
**Up to baseline (mL/kg)**	238.7 (−41.3–725.2)	183.5 (159.7–459.1)	244.2 (−41.3–720.1)	231.1 (−25.4–725.2)	0.9
**Up to Year 1 (mL/kg)**	370.9 (−106.0–2269.8)	335.05 (228.33–627.8)	467.6 (−106.0–2269.8)	323.7 (182.22–946.7)	0.5
**Iron Overload Treatment Regimen**					0.6
**Desferoxamine(%)**	1 (2.8)	0 (0)	0 (0)	1 (9.1)	
**Deferasirox(%)**	32 (91.4)	6 (100)	17 (94.4)	9 (81.8)	
**Deferasirox and Desferoxamine(%)**	1 (2.9)	0 (0)	0 (0)	1 (9.1)	
**Therapeutic Phlebotomy (%)**	1 (2.9)	0 (0)	1 (5.6)	0(0)	
Splenectomy * (%)					0.11
**Yes**	12 (31.6)	3 (42.9)	3 (15.8)	6 (50)	
**No**	26 (68.4)	4 (57.1)	16 (84.2)	6 (50)	

**Notes:** * All were total splenectomy procedures.

**Table 2:** Iron burden status stratified by *GSTM1* genotype information.

Iron Overload Variables and Chelation	Total (*n* = 38)	*GSTM1* Homozygotes (*n* = 7)	*GSTM1* Heterozygotes (*n* = 19)	*GSTM1* Wild Type (*n* = 12)	*p*-Value
R2*MRI LIC (mg/g of Liver Dry Weight)					
**Baseline**	9.2 ± 5.9	7.8 ± 6.9	8.2 ± 4.85	11.4 ± 6.85	0.09
**Year 1**	8.1 ± 5.4	7.9± 4.7	7.2 ± 4.5	9.5± 6.8	0.82
**Mean change**	-0.7 ± 3.8	0.1 ± 3.3	-0.3 ± 3.2	−1.9 ± 4.9	**0.047**
**Serum Ferritin (ng/dL)**					
**Baseline**	2879.7 ± 1528	2413.6 ± 1133.7	2763.6 ± 1284.4	3335.3 ± 2016.	0.29
**Year 1**	3130.2 ± 2012.7	2576.1 ± 1025.5	3184.6 ± 1962.9	3367.2 ± 2546.6	0.71
**Mean change**	250.6 ± 1129.9	162.6 ± 845.4	421.1 ± 1197.5	31.9 ± 1204.5	0.11
MRI Heart T2* (msec)					
**Baseline ^#^**	37.5 ± 6.7	36.9 ± 3.54	37.7 ± 7.6	37.8 ± 7.8	0.86
**Year 1**	38.3± 6.03	40.7± 2.1	38.7 ± 5.8	36.2 ± 7.6	0.31
**Mean change**	0.9 ± 6.0	3.92 ± 2.6	0.95 ± 7.9	−1.2 ± 3.2	0.2
**Duration of chelation (Months)**	17.76 ± 10.4	14.38 ± 11.3	18.01 ± 9.9	19.2 ± 11.4	0.45
**Iron chelation MPR (%) ^##^**	81 ± 16	83 ± 19	82 ± 15	78 ± 17	0.84

**Notes:** Results presented as mean and ±1 SD unless otherwise specified. # Baseline heart T2*MRI assessment was performed in 23 patients (*GSTM1*-null genotype *n* = 5; *GSTM1* heterozygous genotype *n* = 11; *GSTM1* wild type *n* = 7). ^##^ Calculated monthly and averaged for the interval between baseline and Year 1. Only 28 patients have medication possession ratio (MPR) data available. LIC: liver iron content.

## Supplementary Materials

The following are available online at https://www.mdpi.com/2077-0383/8/11/1878/s1, Figure S1: Serum ferritin according to *GSTM1* genotype groups; Figure S2: Heart T2* according to *GSTM1* genotypes.
