# Supplementary material for: GSTM1 and Liver Iron Content in Children with Sickle Cell Anemia and Iron Overload
Source: J Clin Med. 2019 Nov 5;8(11):1878. doi: 10.3390/jcm8111878 (PMC6912836; doi:10.3390/jcm8111878)
Supplement: Supplementary file 1 [file jcm-08-01878-s001.pdf]

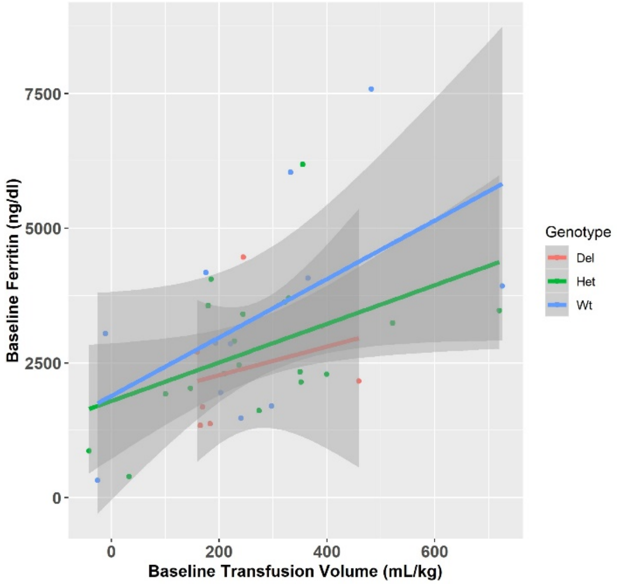

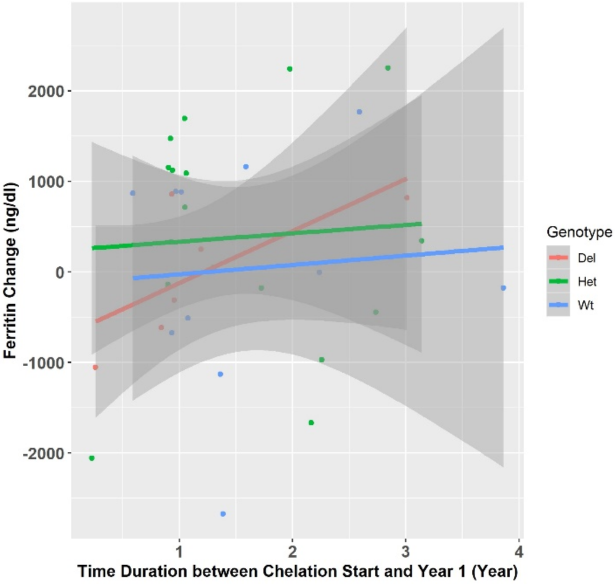

Figure S1a.

Figure S1b

Supplemental

Figure 1. Serum ferritin according to *GSTM1* genotype groups. S1a) Baseline serum ferritin is variable for similar transfusion burden and not significantly different in three genotype groups ($p=0.29$) S1b) Mean change in serum ferritin was not significantly different among the three different genotypes ($p=0.11$) but followed with minimal increase in WT group as compared to *GSTM1* homozygous and heterozygous deletion groups.

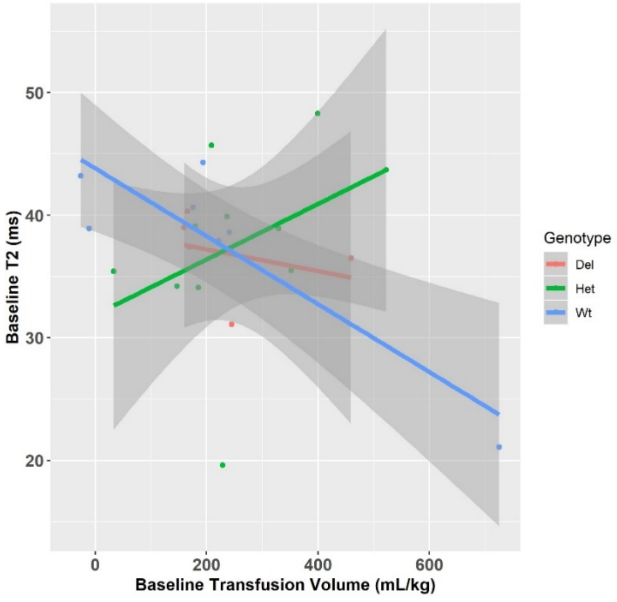

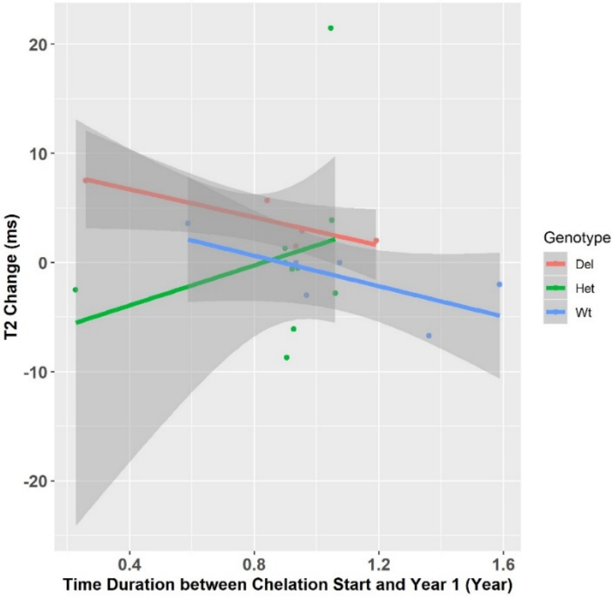

Figure S2a.

Figure S2b.

Supplemental Figure 2. Heart T2* according to *GSTM1* genotypes. S2a) Baseline heart T2* was not significantly different among the three different genotype groups. S2b) Mean change in heart T2* was not significantly different three different genotype groups (*p=0.11*).
